# Natural Polymorphisms in Human APOBEC3H and HIV-1 Vif Combine in Primary T Lymphocytes to Affect Viral G-to-A Mutation Levels and Infectivity

**DOI:** 10.1371/journal.pgen.1004761

**Published:** 2014-11-20

**Authors:** Eric W. Refsland, Judd F. Hultquist, Elizabeth M. Luengas, Terumasa Ikeda, Nadine M. Shaban, Emily K. Law, William L. Brown, Cavan Reilly, Michael Emerman, Reuben S. Harris

**Affiliations:** 1 Department of Biochemistry, Molecular Biology, and Biophysics, University of Minnesota, Minneapolis, Minnesota, United States of America; 2 Institute for Molecular Virology, University of Minnesota, Minneapolis, Minnesota, United States of America; 3 Center for Genome Engineering, University of Minnesota, Minneapolis, Minnesota, United States of America; 4 Masonic Cancer Center, University of Minnesota, Minneapolis, Minnesota, United States of America; 5 Division of Biostatistics, School of Public Health, University of Minnesota, Minneapolis, Minnesota, United States of America; 6 Division of Human Biology, Fred Hutchinson Cancer Research Center, Seattle, Washington, United States of America; University of Wisconsin, United States of America

## Abstract

The Vif protein of HIV-1 allows virus replication by degrading several members of the host-encoded APOBEC3 family of DNA cytosine deaminases. Polymorphisms in both host *APOBEC3* genes and the viral *vif* gene have the potential to impact the extent of virus replication among individuals. The most genetically diverse of the seven human *APOBEC3* genes is *APOBEC3H* with seven known haplotypes. Overexpression studies have shown that a subset of these variants express stable and active proteins, whereas the others encode proteins with a short half-life and little, if any, antiviral activity. We demonstrate that these stable/unstable phenotypes are an intrinsic property of endogenous APOBEC3H proteins in primary CD4+ T lymphocytes and confer differential resistance to HIV-1 infection in a manner that depends on natural variation in the Vif protein of the infecting virus. HIV-1 with a Vif protein hypo-functional for APOBEC3H degradation, yet fully able to counteract APOBEC3D, APOBEC3F, and APOBEC3G, was susceptible to restriction and hypermutation in stable APOBEC3H expressing lymphocytes, but not in unstable APOBEC3H expressing lymphocytes. In contrast, HIV-1 with hyper-functional Vif counteracted stable APOBEC3H proteins as well as all other endogenous APOBEC3s and replicated to high levels. We also found that APOBEC3H protein levels are induced over 10-fold by infection. Finally, we found that the global distribution of stable/unstable APOBEC3H haplotypes correlates with the distribution a critical hyper/hypo-functional Vif amino acid residue. These data combine to strongly suggest that stable APOBEC3H haplotypes present as *in vivo* barriers to HIV-1 replication, that Vif is capable of adapting to these restrictive pressures, and that an evolutionary equilibrium has yet to be reached.

## Author Summary

The APOBEC3 enzymes protect cells by inhibiting the spread of retroelements, including HIV-1, by blocking reverse transcription and mutating cytosines in single-stranded DNA replication intermediates. HIV-1 Vif counteracts restriction by marking APOBEC3 proteins for proteasomal degradation. APOBEC3H is the most diverse member of this protein family. Humans have seven distinct *APOBEC3H* haplotypes with three producing stable and four producing unstable proteins upon forced overexpression. Here, we examine the stability phenotype of endogenous APOBEC3H in donors with different haplotypes and address how these stability differences, as well as natural viral diversity, combine to determine HIV-1 infectivity. We found that endogenous *APOBEC3H* haplotypes yield stable or unstable proteins and that stable APOBEC3H is induced during viral infection and restricts the replication of isolates with naturally occurring hypo-functional but not hyper-functional Vif alleles. We also found that the global distribution of stable APOBEC3H alleles correlates with the prevalence of HIV-1 Vif alleles capable of mediating its degradation, strongly suggesting that the viral Vif protein is capable of adapting to the APOBEC3H restriction potential of an infected individual. Thus, the combination of human APOBEC3H haplotypes and virus Vif alleles may help account for some of the observed disparities in disease progression and virus transmission.

## Introduction

The human APOBEC3 (A3) family of DNA cytosine deaminases is encoded by seven genes arranged in tandem on chromosome 22 (reviewed by [1], [2]). These proteins inhibit the replication of a broad number of parasitic elements, including many retroviruses, some DNA viruses, and several endogenous retroelements and retrotransposons by both deaminase-dependent and -independent mechanisms (reviewed by [2]–[4]). Several lines of evidence indicate that A3D, A3F, A3G, and A3H contribute to HIV-1 restriction by packaging into assembling virus particles and, upon virus entry into new target cells, deaminating viral cDNA cytosines to uracils and impeding the progression of reverse transcription (reviewed by [2]–[4]). These cDNA uracil lesions template the insertion of genomic strand adenines during second strand reverse transcription and ultimately manifest as G-to-A mutations which destroy virus infectivity by the introduction of deleterious missense and nonsense mutations within viral open reading frames.

HIV-1 encodes an accessory protein called Vif (virion infectivity factor) that functions to neutralize cellular A3 proteins. HIV-1 Vif assembles an E3 ligase complex comprised of CBF-β, ELOB, ELOC, CUL5, and RBX2 to mediate the poly-ubiquitination and proteasomal degradation of restrictive A3s ([5], [6] and references therein). This process enables HIV-1 to replicate in its main target cell, CD4+ T lymphocytes, which express multiple A3s and would otherwise be non-permissive for viral replication [7]–[9]. However, this process is less than 100% effective, as G-to-A mutations are commonly observed in viral sequences from clinical specimens and, depending on the patient, may be biased toward a GG-to-AG dinucleotide context indicative of A3G or a GA-to-AA characteristic of A3D, A3F, and/or A3H [7]–[23].

The present day human *A3* locus is a result of multiple gene duplication events during evolution from an ancestral mammalian locus [24]. Unlike the Z1 domain (*e.g. A3A*) or Z2 domain (*e.g. A3C*) deaminase genes, which show considerable copy number variation between mammalian phylogenetic tree branches, the Z3 domain gene (*e.g. A3H*) exists in only one copy in all mammalian genomes sequenced to date [25]. Overexpression studies have shown that A3H and the orthologous Z3 domain deaminases from several mammalian species have a conserved capacity to restrict retrovirus replication, and likewise are neutralized by the Vif proteins of each species' lentivirus [25]–[33]. However, a role for A3H *in vivo* has not yet been elucidated since endogenous expression of the protein has been difficult to detect [34].

*A3H* is the most polymorphic of the human *A3* genes due to circulation of at least 7 distinct *A3H* haplotypes [35], [36]. These haplotypes are comprised of various combinations of 5 single nucleotide polymorphisms located in exons 2, 3 and 4 that range in allele frequencies globally from 6 to 87% [37]. Previous overexpression and pulse-chase experiments have shown that 3 *A3H* haplotypes yield proteins with relatively long half-lives (stable), 1 produces a protein with weak stability, and another 3 make completely unstable proteins [35], [36], [38]. For example, transient transfection of cells with *A3H* haplotype II cDNA with SNPs at residues 15, 18, 105, 121, and 178 (NRRDD) yields a protein readily detectable by immunoblotting, whereas *A3H* haplotypes I (NRGKE) and III (ΔRRDD) produce weakly expressed or undetectable proteins, respectively [35], [36]. However, it is not yet known whether these dramatic haplotype-associated stability/instability phenotypes also manifest for endogenous A3H proteins expressed in primary immune cells. This distinction is important because only stably expressed haplotypes would be predicted to exert selective pressure on HIV-1 replication and potentially contribute to virus restriction and diversification *in vivo*. Indeed, a recent paper linked putatively stable A3H haplotype II to enhanced restriction, higher frequencies of G-to-A mutation, and more favorable clinical phenotypes (lower viral loads and higher patient CD4+ T cell numbers) [39].

Here, we test the hypothesis that HIV-1 infectivity will be influenced by both host *A3H* genotype as well as viral *vif* genotype. We detect the expression and induction of endogenous A3H protein from stable *A3H* alleles following HIV-1 infection. By constructing a set of molecular clones encoding Vif proteins of varying abilities to antagonize stable A3H, we showed that expression of one allele of stable *A3H* is sufficient to inhibit hypo-functional Vif virus replication and promote G-to-A mutagenesis in the expected GA-to-AA dinucleotide context. In contrast, a hyper-functional Vif variant effectively counteracted stable A3H activity. Virus adaptation to stable A3H may be occurring on a global scale because the geographic distribution of a key hyper/hypo-Vif amino acid correlates with the distribution of stable/unstable A3H haplotypes. Taken together, these data demonstrate that stable A3H haplotypes affect HIV-1 replication in the primary target cells of the virus, and further suggest that viral evolution requires the Vif protein to adapt to counteract the restrictive pressure imposed by these enzymes. Therefore, the combination of *A3H* haplotype and viral *vif* variation may be critical and linked factors in HIV-1 adaptation to human populations.

## Results

### At least two A3H haplotypes encode stable proteins in primary T lymphocytes

**Figure 1:**
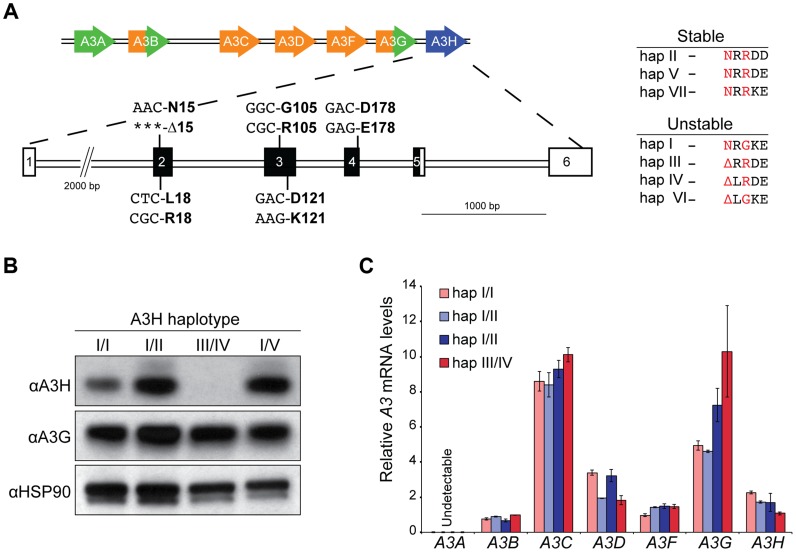
Endogenous APOBEC3H stability/instability occurs at the protein level. A) A schematic of the 7 gene *A3* locus and the 5 polymorphisms in *A3H* exons 2, 3, and 4 that combine to produce 7 different haplotypes. On the right, a summary of the 7 different A3H haplotypes based on observed protein stability or instability in overexpression studies [35], [36] and as defined here, in primary CD4+ T lymphocytes. The two residues that underlie the stable/unstable phenotypes are highlighted in red (N15/Δ15 and R105/G105). B) Immunoblots showing endogenous A3H, A3G, and HSP90 protein levels in stimulated primary T lymphocytes from 4 donors with the indicated *A3H* haplotypes (donors 10, 11, 12, and 18). In this experiment endogenous A3H is detected with a polyclonal rabbit antibody. C) *A3* mRNA levels in primary T lymphocytes from 4 donors with the indicated *A3H* haplotypes (donors 4, 10, 12, and 18). Expression levels are shown relative to the housekeeping gene *TBP*.

To address whether the dramatic protein stability phenotypes observed previously in overexpression studies ([36], [38], [40]; **Figure 1A**) extend to endogenous *A3H* haplotypes in primary T cells, we used our recently developed anti-A3H monoclonal antibodies [34] as well as a new commercial anti-A3H polyclonal antibody to probe protein expression in CD4+ T lymphocytes from healthy donors with different haplotypes (**Materials and Methods**, **Figure 1B** **& Figure S1**). Primary CD4+ lymphocytes were isolated by negative selection, stimulated with IL2 and PHA for 72 hrs, and subjected to immunoblotting. Three of 24 donors were heterozygous for one copy of a predicted stable *A3H* allele, and none were homozygous (6% allele frequency; **Table S1**). Anti-A3H immunoblots demonstrated clear differences in steady-state protein levels, with robust detection of haplotypes II and V, weaker detection of haplotype I, and no detection of haplotypes III and IV (**Figure 1B**). A3G and HSP90 protein levels were expressed similarly between donors.

To confirm the prediction from previous overexpression studies that the observed difference in A3H protein levels is not at the RNA level, RT-qPCR [9] was used to quantify *A3* mRNA levels in primary CD4+ T lymphocytes. Similar *A3H* mRNA levels were observed regardless of haplotype (**Figure 1C**). The mRNA levels of the other *A3* genes were also unrelated to *A3H* genotype, with some minor variation observed between donors (typically less than 2-fold, as reported [8], [9]). Thus, consistent with prior overexpression and pulse-chase experiments to measure protein half-life [35], [36], [38], the large difference between the various *A3H* haplotypes in primary CD4+ cells is most likely due to a protein level mechanism.

### Identification of Vif residues that interact with stable A3H and still antagonize the other A3 proteins expressed in primary T cells

**Figure 2:**
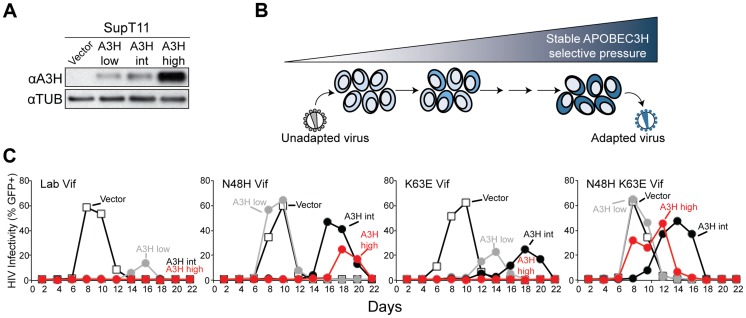
APOBEC3H adaptation studies. A) Immunoblots of A3H and tubulin in SupT11 cell lines stably expressing low, intermediate (int), and high levels of stable haplotype II protein. In this experiment A3H is detected with the mouse monoclonal antibody P3A3-A10. B) Schematic of the stepwise A3H adaptation procedure (see text and **Materials and Methods** for details). C) Spreading infection kinetics of HIV-1 molecular clones encoding the indicated Vif proteins on the A3H-expressing SupT11 lines shown in A.

Primary CD4+ T lymphocytes express 6 *A3* family members [8], [9]. We asked whether or not the ability of Vif to antagonize A3H could be separated from its ability to antagonize the other A3 proteins expressed in primary CD4+ T cells. Previous work indicated that Vif proteins of different HIV-1 isolates show varying capacities to counteract stable A3H (NL4-3/IIIB and LAI [7], [25], [34], [41], [42]), but these studies did not address spreading infections in T cells where A3D, A3F, and especially A3G selective pressures might influence Vif function. To help inform this construction, we performed a series of HIV-1 IIIB N48H adaptation experiments in which this lab-derived strain was subjected by stepwise passage to increasing ratios and levels of stable A3H haplotype II expressed in SupT11 cells (**Figure 2A & B**). An N48H variant was used to initiate these experiments because prior work had already demonstrated that this natural variation improved the A3H counteraction capacity of the Vif protein of IIIB/NL4-3 [42]. These experiments were initiated by infecting (MOI 0.05) uniform pools of stable A3H-low expressing cells (weakly selective) and, after an 8 day incubation, viral supernatants were transferred to the next highest selective condition, and this process was repeated until 100% stable A3H-high conditions were reached (**Materials and Methods**).

**Figure 3:**
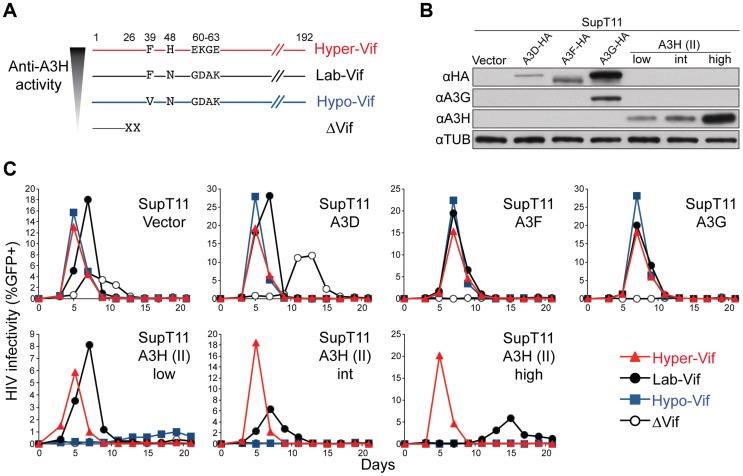
Generation and validation of HIV-1 Vif separation-of-function molecular/viral probes. A) A schematic of the Vif protein encoded by each HIV-1 molecular clone showing amino acid differences responsible for the hyper- and hypo-Vif functionality relative to lab-Vif (HIV-1 IIIB/NL4-3) against stable A3H haplotype II. B) Immunoblots showing the expression levels of the indicated A3 proteins stably expressed in SupT11 cells. In this experiment untagged A3H is detected with the mouse monoclonal antibody P3A3-A10. C) HIV-1 spreading infection kinetics for the indicated viruses on A3-expressing SupT11 cells lines described in panel B. The hyper-, lab-, and hypo-Vif isolates spread with similar kinetics on cells expressing a control vector, A3D, A3F, or A3G, but showed clear phenotypic differences on cells expressing low, intermediate (int), and high levels of stable A3H haplotype II. Delta-Vif virus replication was evident in control vector expressing SupT11 cells, delayed in A3D expressing cells, and suppressed under all other conditions (some symbols eclipsed).

In comparison to the starting virus, HIV-1 IIIB N48H, which only showed fast replication kinetics in SupT11 control vector and A3H-low expressing cells, one of the adapted viruses gained the capacity to replicate quickly in SupT11 cells expressing high levels of stable A3H. The majority (5/9) of *vif* sequences from this adapted population encoded a K63E amino acid substitution. Spreading infection experiments with an isogenic set of molecular clones showed that K63E combined with N48H to improve virus replication even in the presence of high levels of stable A3H (**Figure 2C**). Furthermore, we noticed that the K63E substitution recovered in these adaptation experiments is part of a cluster of 4 amino acids that distinguishes the IIIB lab strain from an isolate recovered from a homozygous stable A3H haplotype II patient [34]. The Vif protein from this particular isolate is even better than LAI Vif at counteracting stable A3H haplotype II [34]. We therefore predicted that the combination of N48H (to be more LAI Vif-like [42]) and GDAK60-63 to EKGE60-63 (to be more haplotype II patient Vif-like [34]) would result in a hyper-functional Vif protein capable of fully antagonizing stable A3H haplotype II (hyper-Vif; **Figure 3A**). In addition, the separation-of-function approach required the generation of a Vif variant even more sensitive than lab-Vif to restriction by stable A3H haplotype II. This was done by introducing F39V into the HIV-1 IIIB lab strain, which is a substitution that confers sensitivity to stable A3H haplotype II [41] (**Figure 3A**).

The A3 neutralization activities of the hyper-, lab-, and hypo-Vif HIV-1 IIIB variants were assessed by comparing spreading infection kinetics in SupT11 cell lines stably expressing a control vector, A3D-HA, A3F-HA, A3G-HA, and low, intermediate, and high levels of untagged A3H haplotype II (**Figures 3B, 3C** **& S2**). The A3 expression level in each SupT11 clone was sufficient to delay (A3D) or fully suppress (A3F, A3G, A3H haplotype II) replication of a Vif-null HIV-1 IIIB control virus, consistent with our original studies using these stable cell lines [7]. Importantly, the hyper-, lab-, and hypo-Vif HIV-1 IIIB variants spread with nearly identical efficiencies in the SupT11 cell lines expressing the control vector, A3D-HA, A3F-HA, and A3G-HA, demonstrating fully intact capacities to neutralize these restriction factors. In contrast, each isolate exhibited differential replication kinetics on the SupT11 lines expressing low, intermediate, and high levels of stable A3H haplotype II. The hyper-Vif isolate replicated with strong kinetics under all conditions including the highest stable A3H haplotype II expression levels, the lab-Vif isolate was delayed under intermediate and high A3H expression conditions, and the hypo-Vif isolate showed restricted replication phenotypes almost indistinguishable from the Vif-deficient control. These data demonstrate that naturally occurring amino acid residues that positively or negatively influence Vif-mediated neutralization of A3H can do so without impacting Vif activity against A3D, A3F, and A3G and therefore allow for a direct interrogation of endogenous A3H function in primary T lymphocytes.

### HIV-1 restriction by stable A3H in primary T lymphocytes

**Figure 4:**
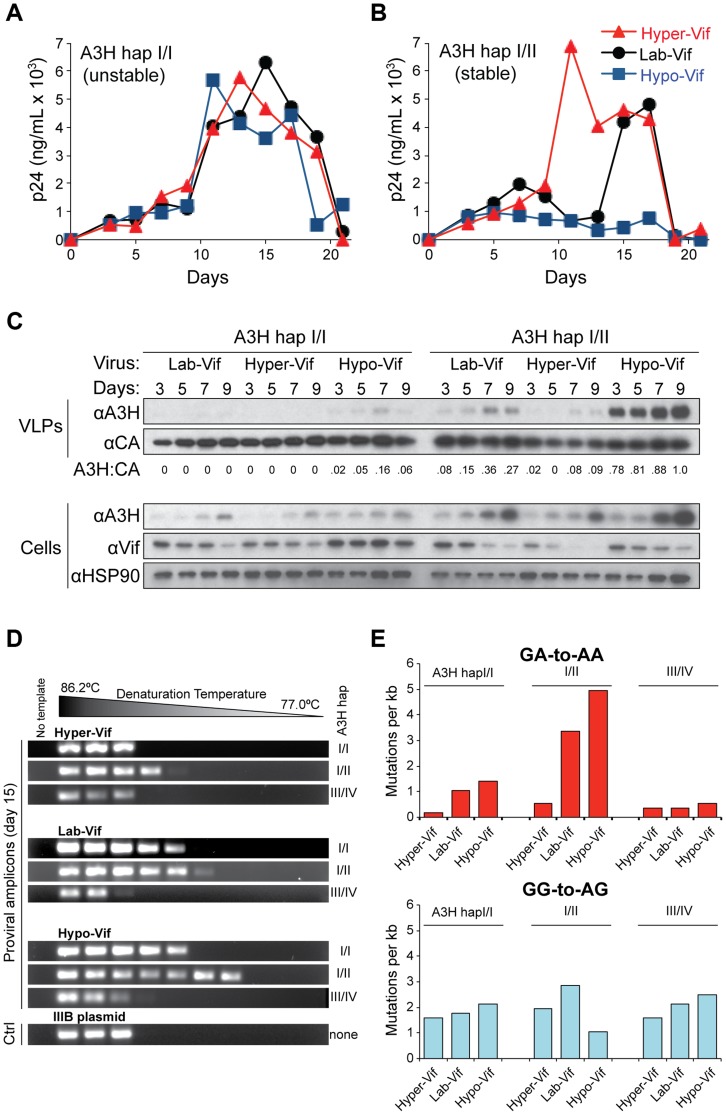
Stable APOBEC3H inhibits HIV-1 replication in primary T lymphocytes and inflicts GA-to-AA hypermutations. A) HIV-1 replication kinetics of the hyper-, lab-, and hypo-Vif variants in CD4+ T lymphocytes from a representative healthy donor encoding unstable A3H haplotype I/I (donor 2). Data from additional experiments using independent donors are shown in Figure S3. Y-axis values represent p24 levels measured by ELISA. B) HIV-1 replication kinetics of the hyper-, lab-, and hypo-Vif variants in CD4+ T lymphocytes from a representative healthy donor encoding one allele of stable A3H haplotype II (and one allele of unstable A3H haplotype I; donor 4). Data from additional experiments using independent donors are shown in Figure S3. Y-axis values represent p24 levels measured by ELISA. C) Immunoblots of the indicated proteins in non-normalized virus-like particle (VLP) supernatants and in cells from days 3, 5, 7, and 9 of the spreading experiments shown in panels A & B. A quantification of the ratio of A3H to CA is shown below each VLP blot set. D) 3D-PCR amplicons generated from proviral DNA of the indicated viruses isolated on day 15 of a spreading infection of unstable A3H (haplotypes I/I and III/IV) or stable A3H (haplotype I/II) donor cells (donors 2, 4, 12). This experiment is representative and performed independently of those shown in panels A-C. E) Histograms depicting the frequencies of GA-to-AA and GG-to-AG mutations under the indicated spreading infection conditions (complementary to the experiment shown in panel D with all sequences derived from independent 98°C high-fidelity PCR amplifications). A minimum of 10 clones were sequenced for each condition (≥5 kb).

To determine the relative importance of the polymorphisms of A3H and Vif on HIV-1 replication in primary cells, a series of spreading infection experiments was initiated in CD4+ cells from individuals who encode different haplotypes of A3H with viruses encoding the hyper-, lab-, and hypo-Vif alleles that differentially antagonize A3H. We found that all 3 viruses replicated with similar kinetics in primary CD4+ T cells from donors homozygous for unstable A3H haplotype I or heterozygous for unstable A3H haplotypes III and IV. Indicating that hyper-, lab-, and hypo-Vif viruses are all capable of neutralizing the restrictive levels of A3D, A3F, and A3G expression in primary cells (**Figure 4A** **& S3**). In contrast, only the hyper-Vif virus replicated with robust kinetics in CD4+ T cells from donors heterozygous for stable A3H haplotypes II or V (**Figure 4B** **& S3**). The lab-Vif and hypo-Vif viruses showed delayed and strongly inhibited replication kinetics, respectively, in CD4+ T cells from donors heterozygous for stable A3H haplotypes (**Figure 4B** **& S3**). All of the heterozygous stable A3H haplotype II donors also had a copy of unstable A3H haplotype I, so these large differences in replication kinetics are due to only a single stable A3H allele. Thus, these experiments demonstrate that even a single allele of endogenous stable A3H constitutes a formidable barrier to replication of susceptible HIV-1 isolates.

The APOBEC3 proteins must be packaged into viral particles to restrict HIV-1 replication. To confirm that stable, endogenous A3H acts by this mechanism, aliquots of cells and virus-containing supernatants were taken on days 3, 5, 7 and 9 post-infection for each donor/virus condition and analyzed by immunoblotting (**Figure 4C**). First, we observed that A3H is induced at the protein level over the course of infection. This is most evident in cells infected with the hypo-Vif virus, most likely because this Vif variant fails to bind endogenous A3H and trigger its degradation. The level of A3H induction observed here at the protein level corresponds roughly to the level of induction observed previously at the mRNA level (>10-fold [7]). Second, there is a strong correlation between cellular Vif, cellular A3H, and packaged A3H levels. This is most evident upon direct comparison of the hyper-Vif and hypo-Vif infections. For the hyper-Vif infection of stable haplotype II cells, Vif disappears, cellular A3H is induced weakly, and very little viral A3H is observed in particles. In contrast, for the hypo-Vif infection of stable haplotype II cells, Vif decreases modestly (consistent with its role in mediating the degradation of A3D, A3F, and A3G), cellular A3H is induced strongly, and large amounts of A3H are observed in particles. As expected from the SupT11 experiments described above, the lab-Vif spreading infection elicited intermediate phenotypes. Most importantly, levels of A3H in viral particles correlated with virus replication kinetics, with the hyper-Vif virus with low A3H incorporation spreading robustly and the hypo-Vif virus with high A3H incorporation being restricted almost completely. Thus, HIV-1 replication, as well as the induction and encapsidation of A3H, are influenced by both host *A3H* haplotype and the viral *vif* genotype.

### G-to-A mutation spectra of Vif separation-of-function isolates subjected to different endogenous A3H haplotypes

The hallmark activity of HIV-1-restrictive A3 family members is viral cDNA deamination of C-to-U, with the uracil lesions being converted into genomic strand G-to-A mutations (reviewed by [3], [4], [43], [44]). To determine the correlation between mutagenesis and the relative restriction incurred by each of the separation-of-function Vif variants in combination with different A3H haplotypes, the viruses from each stable and unstable A3H experimental condition were subjected to analysis by differential DNA denaturation (3D)-PCR [7], [45], [46]. This technique depends on the fact that every PCR amplicon has a characteristic denaturation temperature that must be reached in order to yield visible PCR product, and G-to-A mutations within the amplicon will result in the appearance of product at significantly lower temperatures. After 15 days of spreading infection, viral supernatants were removed and used to infect CEM-GFP reporter cells. After allowing 2 days for infection and integration, total cellular DNA was prepared, quantified, and subjected to 3D-PCR analysis of a 564 bp HIV-1 *pol* region (**Figure 4D**). All viruses from unstable A3H haplotype III/IV expressing cells showed no evidence for G-to-A hypermutation, as the lowest denaturation temperature with visible PCR product was the same as that of the original molecular clone. The level of G-to-A mutation in hypo-Vif viruses from stable A3H haplotype II/I cells was clearly the highest, but levels for lab-Vif and hyper-Vif viruses were also significantly above the background level established by the amplification cut-off for the original molecular clone. Interestingly, a significant level of G-to-A mutation was also evident in hypo-Vif and lab-Vif viruses (but not in hyper-Vif virus) from unstable haplotype I/I cells, indicating that A3H haplotype I protein is capable of lower levels of viral cDNA deamination and may contribute to HIV-1 mutagenesis, though not to obvious infectivity decreases.

To analyze the mutation spectrum and local dinucleotide mutation contexts, we used normal high-fidelity PCR (98°C denaturation temperature) and Sanger sequencing to analyze the *pol* region of proviral DNA from an independent experiment (*i.e.*, viruses from day 15 of the experiment shown in **Figure 4A–C**). Consistent with the 3D-PCR experiment discussed above, the highest level of G-to-A mutation occurred in hypo-Vif viruses from cells expressing stable A3H haplotype II, and the lowest level of G-to-A mutation occurred in hyper-Vif viruses from cells expressing unstable A3H haplotype I (5.0 G-to-A mutations per kb versus 0.2 G-to-A mutations per kb; **Figure 4E**). Most (76%) of the hypo-Vif viruses' G-to-A mutations occurred in a GA-to-AA context, consistent with the 5′TC deamination preference of A3H shown previously [7], [39], [47]. In contrast, few G-to-A mutations were observed in a GG-to-AG context characteristic of A3G, further supporting the SupT11 experiments shown above that demonstrate that each of the Vif separation-of-function variants retains equivalent and strong A3G antagonism (**Figure 4E** **& S4**).

### Global correlations between HIV-1 Vif genotypes and human A3H haplotypes

**Figure 5:**
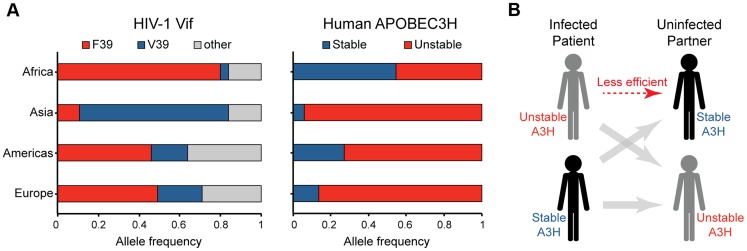
Correlations between the global distributions of HIV-1 hyper-Vif alleles and human A3H haplotypes. A) The left histogram depicts the frequency of HIV-1 isolates encoding a phenylalanine or valine at Vif residue 39 from the indicated geographic regions (n = 9713; www.hiv.lanl.gov). The right histogram shows the frequency of stable versus unstable *A3H* alleles from the same geographic regions (n = 1092; www.1000Genomes.org). B) A model depicting the anticipated relative transmission efficiencies between infected patients and uninfected individuals with equivalent or different *A3H* haplotypes.

If A3H exerts selective pressure on HIV-1, then Vif variants most capable of neutralizing its activity should predominate in areas where stable A3H haplotypes also predominate. A geographic breakdown of 9713 HIV-1 isolates represented in the Los Alamos database revealed that F39 (an A3H resistance residue) predominates in Africa, whereas V39 (an A3H susceptibility residue) predominates in Asia (**Figure 5A** **& Table S2**). In addition, the N48 residue in Vif that confers sensitivity to high levels of stable A3H haplotype II [7], [41], [42] in HIV-1 IIIB and NL4-3, is present in only 9% of African isolates but in 30% of isolates from Asia. Interestingly, these Vif allelic distributions correspond to the worldwide estimates for A3H haplotypes from the 1000 genomes project previously reported [35], [48], with stable haplotypes predominating in Africa and unstable haplotypes in Asia (**Figure 5A** **& Table S3**). The estimated relative risk for having the Vif variant most capable of neutralizing A3H given the stable A3H genotype relative to the unstable genotype is 2.0 with a 95% credible set of (1.7, 2.4). Thus, HIV-1 Vif appears to have the capacity to adapt to the A3H haplotype of a population. A major prediction is therefore that stable A3H may be a protective factor in HIV-1 acquisition, especially in instances in which a stable A3H haplotype individual is exposed to a hypo-Vif inoculum from an unstable A3H haplotype patient (**Figure 5B**).

### A3H-altering Vif residues define an interaction surface

**Figure 6:**
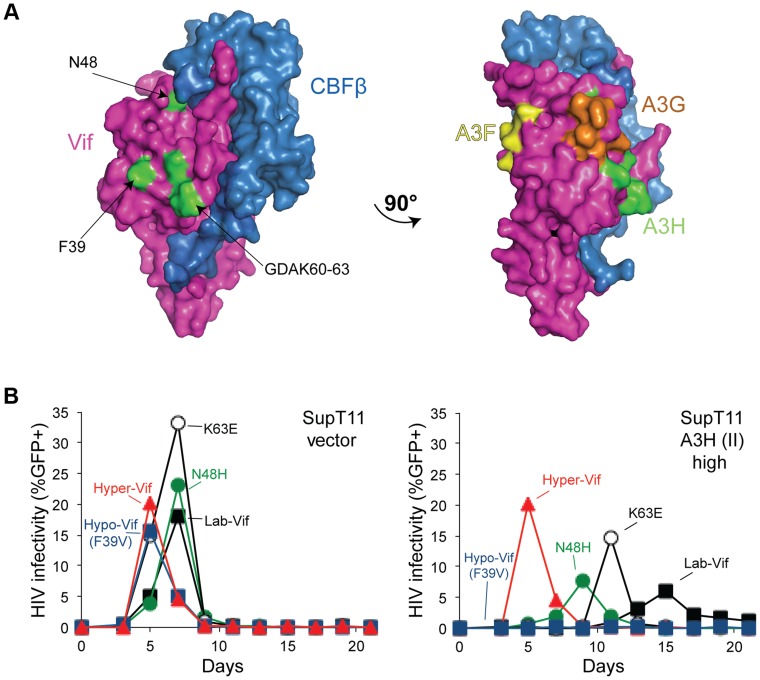
Vif separation-of-function substitutions define the likely APOBEC3H interaction surface. A) A surface representation of Vif and CBFβ (pdb 4N9F). The side-chains of the amino acid residues conferring hypo-Vif (residue 39) and hyper-Vif phenotypes (residues 48 and 60–63) are shaded green and located on a common solvent-exposed surface. A 90° rotation reveals distinct Vif separation-of-function residues implicated in the interactions with A3F (yellow) and A3G (orange). See main text for details. B) Spreading infection kinetics of the indicated HIV-1 Vif variants on SupT11 cells stably expressing a vector control (left) or high levels of A3H haplotype II (right). Figure 4C data for hyper-, lab-, and hypo-Vif are shown again here to facilitate comparisons.

All current evidence indicates that Vif heterodimerizes with CBF-β, directly binds restrictive A3s, and recruits an E3-ligase ubiquitin complex to target them for proteasomal degradation [3], [4], [43], [44]. Recently, a crystal structure of the Vif-CBF-β-ELOB-ELOC-CUL5 complex was determined, providing long-awaited details of the molecular architecture of Vif [49]. The separation-of-function Vif variants described here provide an opportunity to define the A3H interaction surface. Interestingly, all of the amino acids that comprise these variants map to the same solvent-exposed surface of Vif (**Figure 6A**). The critical hypo- and hyper-Vif residues, F39 and GDAK60-63, are particularly close together. Moreover, the delayed and differential spreading infection phenotypes of HIV-1 isolates with each of these single amino acid substitutions in SupT11 T cells expressing high levels of stable A3H haplotype II indicates that each of these residues makes partial contribution to the overall hyper-Vif phenotype (**Figure 6B**).

Analogous but physically distinct separation-of-function Vif mutants have been described for A3F and A3G. For instance, the DRMR14-17 motif is required for A3F degradation, but does not affect A3G neutralization [50], [51]. Furthermore, another motif, YRHHY40-44, specifically compromises the A3G interaction [51]. The fact that the A3H interaction surface defined above and these A3F- and A3G-specific motifs map to distinct solvent exposed surfaces strongly indicates that Vif may use different binding modes in order to neutralize each of these restriction factors.

## Discussion

A3H is the most polymorphic A3 family member in the human population, and here we provide the first protein-level demonstration that the differential stability and inducibility of endogenous A3H haplotypes in primary T cells impacts HIV-1 replication in its normal target cells. We also report that natural variation in HIV-1 Vif results in the differential neutralization of stable A3H haplotype II and influences virus replication and hypermutation activities in primary CD4+ lymphocytes. The hyper-Vif virus showed strong replication kinetics and low levels of G-to-A mutation under all conditions tested, whereas the hypo-Vif virus was only restricted and hypermutated in primary CD4+ T lymphocytes expressing endogenous levels of stable A3H haplotype II. Because the separation-of-function variants were based on naturally occurring HIV-1 amino acid residues, our data combine to indicate that stable haplotypes of endogenous A3H may constitute a replication barrier to a significant subset of circulating HIV-1 strains. This conclusion is supported by significant correlations between A3H haplotype stability and predicted Vif functionality in worldwide human and HIV-1 genotype information (p<0.001; **Figure 5** **and Tables S2 & S3**).

Homology to other A3s with high-resolution structural information indicates that N15 is a highly conserved residue near the end of α-helix 1 and therefore most likely required for A3H structural integrity. NMR and mutagenesis studies with the catalytic domain of A3G have shown that this helix is essential for the stability and integrity of the entire deaminase domain, as it makes several essential contacts with internal residues [52], [53]. For instance, an alanine substitution of the homologous A3G residue N208 renders the enzyme catalytically dead [52]. In contrast, the glycine at position 105 is predicted to be in the middle of β-strand 4, located far from N15 and on the backside of the enzyme relative to the catalytic zinc-coordinating active site. This location and the observed intermediate stability of A3H haplotype I protein combine to suggest a different mechanism, possibly by altering an interaction with a cellular binding partner [34]. Consistent with these predictions, all evidence indicates that *A3H* haplotypes III and IV (ΔN15) encode complete loss-of-function proteins, whereas haplotype I (G105) encodes a hypofunctional variant because higher-than-background levels of G-to-A mutations are observed in the hypo-Vif virus in haplotype I expressing T cells.

Our data agree with the main conclusion of a study published recently by the Simon group [39]. They concluded that A3H haplotype II is a relevant HIV-1 restriction factor by showing more G-to-A mutations accumulating in viruses replicating in A3H haplotype II donor PBMC, in comparison to the same viruses replicating in haplotype I donor PMBC [39]. Our studies help explain their results by showing that stable A3H haplotypes are indeed expressed at the protein level in primary HIV-1 target cells and that, depending upon the functionality of the infecting virus's Vif protein, the outcomes can range from aphenotypic (hyper-Vif) to strong infectivity restriction and hypermutation (hypo-Vif). Importantly, the wide variation in G-to-A hypermutation levels observed in our controlled experiments may help explain the similarly wide variation reported in patient derived HIV-1 sequences [7]–[23]. Variation occurs for both overall G-to-A mutation frequency and for local dinucleotide preferences with, in many instances, GA-to-AA mutations predominating (*e.g.* [10], [11]). These *in vivo* biases could be due to the combination of stable A3H haplotypes and infection by hypo-functional Vif isolates.

The results presented here, together with recent data from the Simon group [39], combine to indicate that stable A3H haplotypes may be functioning as contemporary HIV-1 restriction factors. Stable A3H haplotypes may contribute to disease progression by limiting HIV-1 replication within an infected individual, as indicated by statistically lower viral loads and higher CD4+ T cell counts in haplotype II patients in comparison to haplotype I patients [39]. However, stable A3H haplotypes may also affect rates of transmission. In particular, our studies predict lower rates of virus acquisition when an infected patient has an unstable haplotype and their uninfected partner has a stable A3H haplotype (**Figure 5B**). Moreover, in instances where HIV-1 breaches this transmission barrier, our studies predict that the Vif protein will have to adapt in order to effectively counteract stable A3H. Thus, HIV-1 may need to adapt differently to the A3 repertoire in different humans, supported by correlations between worldwide Vif and A3H genotypes (p<0.001; **Figure 5** **and Tables S2 & S3**). Overall, polymorphisms in human A3H and HIV-1 Vif appear to be combining to actively limit HIV-1 pathogenesis.

## Materials and Methods

### Virus constructs

Vif-proficient (GenBank EU541617) and Vif-deficient (X26, X27) HIVIIIB A200C proviral constructs have been described [54], [55]. Substitutions to construct the hypo-Vif and hyper-Vif variants were introduced with site-directed mutagenesis using primers RSH6951 5′TCA-AGG-AAA-GCT-AAG-GAC-TGG-GTT-TAT-AGA-CAT-CAC-TAT-GAA-AG & RSH6952 5′CTT-TCA-TAG-TGA-TGT-CTA-TAA-ACC-CAG-TCC-TTA-GCT-TTC-CTT-GA for the F39V substitution, RSH6949 5′TTT-ATA-GAC-ATC-ACT-ATG-AAA-GTA-CTC-ATC-CAA-AAA-TAA-GTT-CAG-AAG-TAC-AC & RSH6950 5′GTG-TAC-TTC-TGA-ACT-TAT-TTT-TGG-ATG-AGT-ACT-TTC-ATA-GTG-ATG-TCT-ATA-AA for the N48H substitution, and RSH6965 5′TCC-AAA-AAT-AAG-TTC-AGA-AGT-ACA-CAT-CCC-ACT-AGA-GAA-GGG-CGA-GTT-AGT-AAT-AAC-AAC-ATA-TTG-GGG-TCT-GCA-TAC-AGG & RSH6966 5′CCT-GTA-TGC-AGA-CCC-CAA-TAT-GTT-GTT-ATT-ACT-AAC-TCG-CCC-TTC-TCT-AGT-GGG-ATG-TGT-ACT-TCT-GAA-CTT-ATT-TTT-GGA for the GDAK60-63EKGE substitutions.

### Cell lines

SupT11 and CEM-GFP T cells were maintained in RPMI supplemented with 10% fetal bovine serum (FBS) and 0.5% penicillin/streptomycin (P/S). 293T cells were cultured in DMEM supplemented with 10% FBS and 0.5% P/S. The generation and characterization of the SupT11 panel stably expressing vector, A3D-HA, A3F-HA, A3G-HA, and untagged A3H haplotype II have been described [7].

### APOBEC3H antibodies and immunoblotting

APOBEC3H monoclonal antibodies P1H6-1, P1D8-1, P3A1-1, and P3A3-A10 were generated previously [34] and purified by ammonium sulfate precipitation. We presume that the increased sensitivity of these antibodies over the original description [34] is due to purification and increased concentration relative to the unpurified culture supernatants used previously. Aliquots of two of these monoclonal antibodies have been made available through the NIH AIDS Reagent Program as #12155 (P3A3-A10) and 12156 (P1H6-1). Specificity was demonstrated by immunoblotting lysates from 293T cells transiently transfected with HA-tagged A3A, A3B, A3C, A3D, A3F, A3G, and A3H haplotype II (**Figure S1A**). Epitopes were mapped by immunoblotting lysates from 293T cells transiently transfected with a panel of chimeric human/cow A3H/A3Z3 constructs (**Figure S1C**). A polyclonal antibody raised against APOBEC3H residues 45–183 was used according to the manufacturer's instructions (NBP1-91682, Novus Biologicals). Cells were pelleted, washed, and then directly lysed in 2.5X Laemmli sample buffer. Virus containing supernatants were filtered and virus-like particles isolated by centrifugation through a 20% sucrose cushion and resuspended in 2.5X Laemmli sample buffer. Lysates were subjected to SDS-PAGE and protein transfer to PVDF using the Criterion system (Bio-Rad).

### RT-qPCR

Isolation of total RNA, reverse transcription, and qPCR were performed as described [9]. Briefly, total RNA was isolated from cells using the RNeasy kit (Qiagen). 1 µg of RNA was used to generate cDNA with Transcriptor reverse transcriptase (Roche) according to the manufacturer's instructions using random hexameric primers. Quantitative PCR was performed using a LightCycler480 instrument (Roche). All reactions were done in triplicate and *A3* levels were normalized to the housekeeping gene *Tata Binding Protein* (*TBP*).

### Selection of APOBEC3H adapted viruses

The procedures to adapt viruses in culture to an APOBEC3 challenge are based on prior reports [54], [55]. HIV-1 IIIB Vif N48H was generated as described above. The selection experiments were initiated at a MOI of 0.05 by infecting 150,000 cells, 100% of which were SupT11 expressing low levels of A3H haplotype II in a total volume of 1 ml in one well of a 24 well plate. Infections were monitored by removing supernatants and infecting the reporter cell line CEM-GFP every 3–4 days. The infected cells were split and the media was replenished at 4 days post-infection to prevent overgrowth of the culture. On day 8 of the infection, 250 µl of the virus containing supernatant from the infected wells was passaged to 150,000 SupT11 cells expressing higher levels of A3H haplotype II (50% low/50% intermediate). The virus supernatants were passaged in a stepwise manner 4 additional times into cultures with increasing levels of A3H haplotype II expression: 1∶2∶1 low∶intermediate∶high; 1∶1 int∶high; 1∶3 int∶high; 100% high. To purify emerging adapted viruses, a culture of 100% SupT11 expressing high levels of A3H haplotype II was infected at a MOI of 0.05 an additional 3 times. Finally, viruses were used to infect CEM-GFP and genomic DNA was isolated using the Puregene reagents (Qiagen). Proviral DNA encoding a 1287-bp fragment of *pol-vif-vpr* amplified with primers RSH1438 5′CCC-TAC-AAT-CCC-CAA-AGT-CA and RSH1454 5′ CAA-ACT-TGG-CAA-TGA-AAG-CA. Amplicons were cloned into CloneJet (ThermoScientific) and Sanger sequenced using flanking plasmid-specific primers.

### HIV-1 spreading infections

Vif-proficient and Vif-deficient HIV-1 spreading infections were performed as previously described [54]. Viruses were generated by transfecting 10 µg of proviral expression construct into 293T cells. Titers of the viruses were assessed using the CEM-GFP reporter cell line. SupT11 spreading infections were initiated at a 0.01 MOI and the infection was monitored every 2–3 days using the CEM-GFP reporter line [56]. Primary CD4+ T cells were infected at a 0.02 MOI and culture supernatants were collected every 2–3 days and analyzed using an in-house p24 ELISA. Supernatants from infected cultures were incubated with for 1 hr on anti-p24 mAb (183-H12-5C, NIH ARRRP) coated 96-well plates (Nunc). Following 4 washes with PBS 0.1% Tween 20 (PBS-T), a second 1 hr incubation anti-p24 mAb (9725) was used to ‘sandwich’ the p24 antigen. Wells were again washed 4 times, p24 was quantified by 0.5 hr incubation with an enzyme-linked secondary goat anti-mouse IgG-2A/HRP followed by 4 PBS-T wash steps and incubation with 3,3′,5,5′ tetramethybenzidine (TMB) for 6 min. The reaction was stopped upon addition of 1 M H_2_SO_4_ and absorbance at 450 nm was quantified on a microplate reader (Synergy MX, Biotek).

### Primary cells

Peripheral blood mononuclear cells were isolated from whole blood (Memorial Blood Center, St. Paul, MN) by ficoll gradient centrifugation as previously described [7], [9]. Naïve CD4+ T lymphocytes were purified by negative selection according to the manufacturer's instructions (Miltenyi Biotech). Cells were stimulated and maintained in RPMI with 20 U/mL human interleukin-2 (Miltenyi Biotech) and 10 µg/ml phytohemagglutinin (Thermo Scientific). Cells were stimulated for 72 hours prior to infection or harvesting for total RNA and protein lysate. Purity (>95%) and activation were confirmed by staining with an anti-CD4 antibody or anti-CD25 antibody respectively (Miltenyi Biotech).

### Ethics statement

This study has been reviewed and exempted by the University of Minnesota Institutional Review Board (#0503E68687). All PBMCs were isolated from healthy and de-identified individuals.

### *APOBEC3H* genotyping

Primers and PCR conditions for polymorphisms (N15/ΔN15A3H, rs140936762; R18/L18, rs139293; G105/R105, rs139297; D121/K121, rs139299 & rs139298; E178/D178, rs139302) genotyping have been described [36]. Amplicons were cloned into CloneJet (ThermoScientific) and Sanger sequenced. When possible, linkage of heterozygous polymorphic sites was established by Sanger sequencing of amplicons from cDNA following reverse transcription of total RNA. Healthy donors were assessed for A3H haplotype by preliminary screening of PBMCs by immunoblot for A3H protein expression and a diagnostic PCR for the N15/ΔN15 polymorphism. CD4+ T cells were isolated from the selected donors (**Table S1**) as described above.

### 3D-PCR

To assess global hypermutation semi-quantitatively, 3D-PCR was performed as described [7]. 15 days postinfection virus containing supernatants were used to infect 25,000 CEM-GFP cells. 48 hours later, genomic DNA was isolated (Qiagen) and an 876 bp region of *pol* was amplified from proviral DNA. The relative amount of this amplicon in each sample was assessed with qPCR (Roche). Normalized amounts of proviral DNA was then used to generate a smaller 564 bp inner amplicon over a range of denaturation temperatures (77.0–86.2°C). The resulting PCR products were fractionated using agarose gels and visualized following ethidium bromide staining.

### Proviral sequencing

Hypermutation spectra analysis was performed as described [7], [46]. The 876 bp outer amplicon generated from proviral *pol* DNA was used to seed a second, inner PCR reaction with a uniform denaturation temperature of 98°C. The resulting 564 bp amplicon was cloned into CloneJet (ThermoScientific). A total of 10 independent clones (>5 kb) were Sanger sequenced for each condition. Clones with identical mutations were eliminated.

### Statistical methods

To test for an association between Vif and the A3H haplotype, the relative risk for having the Vif F39 genotype as it depends on the stable versus unstable A3H haplotype was estimated using the data in Tables S2 and S3, focusing on Asia and Africa and ignoring subjects without these genotypes (*i.e.*, the “Other” category in Table S2). As each of these tables only has data for subjects on 2 of the 3 variables (*i.e.*, either geographic location and A3H or geographic location and Vif genotypes), a data augmentation strategy was employed to estimate the association of interest. The data from both tables were modeled as partially observed multinomial observations with 8 possible outcomes (since there are 3 dichotomous variables). The unknown parameters in this model are the collection of 8 probabilities that determine which outcome was observed and the missing genotype values for either A3H or Vif. Inference was conducted using the Gibbs sampler via a custom C++ program. Ten thousand samples were generated by the Markov chain, the first half of the samples were discarded and every tenth sample was saved to conduct inference. A vague prior distribution was employed for the class probabilities (a Dirichlet prior with all parameters in the prior set to 1). From the set of sampled class probabilities one can obtain the marginal probabilities by summing over the margins and these can be used to calculate the relative risk and obtain a 95% confidence interval for the relative risk. This interval was (1.7, 2.4) with a median value of 2 indicating that the probability of having Vif F39, an A3H resistance residue, is twice as likely if one has the stable A3H haplotype. This relative risk was never less than 1 in all samples, leading to the conclusion that the probability that relative risk is less than 1 is <0.001.

## Supporting Information

Figure S1 Characterization of mouse monoclonal antibodies specific to human APOBEC3H. A) Immunoblots demonstrating the specificity of the A3H monoclonal antibodies P1D8-1 and P3A3-A10 in 293T cells transiently expressing the indicated A3-HA protein. B) Immunoblots of A3H and tubulin expression levels in primary T lymphocytes 9 days after infection with Vif proficient or deficient viruses. A3D-HA expressed poorly in this experiment (donor 25). C) Schematics of human A3H hap II (open box), cow A3Z3 (black box), and A3H/A3Z3 chimeric derivatives. The epitopes for mouse monoclonal antibodies P1D8-1 and P3A3-A10 are shown. Immunoblots of 293T cells transiently transfected with the indicated human/cow chimeric A3H/A3Z3 constructs. Monoclonal antibodies P1D8-1 and P3A3-A10 recognize distinct N- and C-terminal epitopes, respectively.
(TIF)

Figure S2 HIV-1 Vif separation-of-function molecular/viral probes. HIV-1 spreading infection kinetics for the indicated viruses on A3-expressing SupT11 cells lines described in Figure 3B. Data are reproduced here from Figure 3C but plotted on a common Y-axis scale.
(TIF)

Figure S3 Stable APOBEC3H inhibits HIV-1 replication in primary T lymphocytes. A) HIV-1 replication kinetics of the hyper-, lab-, and hypo-Vif variants in CD4+ T lymphocytes from 5 healthy donors encoding the unstable A3H haplotype indicated (donors 1, 5, 8, 12, and 18). B) HIV-1 replication kinetics of the hyper-, lab-, and hypo-Vif variants in CD4+ T lymphocytes from 2 healthy donors heterozygous for the indicated allele of stable A3H (donors 10 and 11). We were not able to identify a second haplotype V donor and therefore have not been able to determine whether the lab-Vif phenotype of donor 11 is reproducible.
(TIF)

Figure S4 Stable APOBEC3H alleles inflict GA-to-AA hypermutations in viruses encoding hypo-Vif variants. HIV-1 G-to-A mutation profiles of the hyper-, lab-, and hypo-Vif proviruses originating from primary T lymphocytes with the indicated A3H haplotype (donors 2, 4, and 12). GA-to-AA mutations characteristic of A3H activity are shown in red.
(TIF)

Table S1 Selected donor genotyping results.
(TIF)

Table S2 Worldwide *vif* allele frequencies.
(TIF)

Table S3 Worldwide *APOBEC3H* haplotype frequencies.
(TIF)
