# Supplementary figures and images for: Natural Polymorphisms in Human APOBEC3H and HIV-1 Vif Combine in Primary T Lymphocytes to Affect Viral G-to-A Mutation Levels and Infectivity
Source: PLoS Genet. 2014 Nov 20;10(11):e1004761. doi: 10.1371/journal.pgen.1004761 (PMC4238949; doi:10.1371/journal.pgen.1004761)

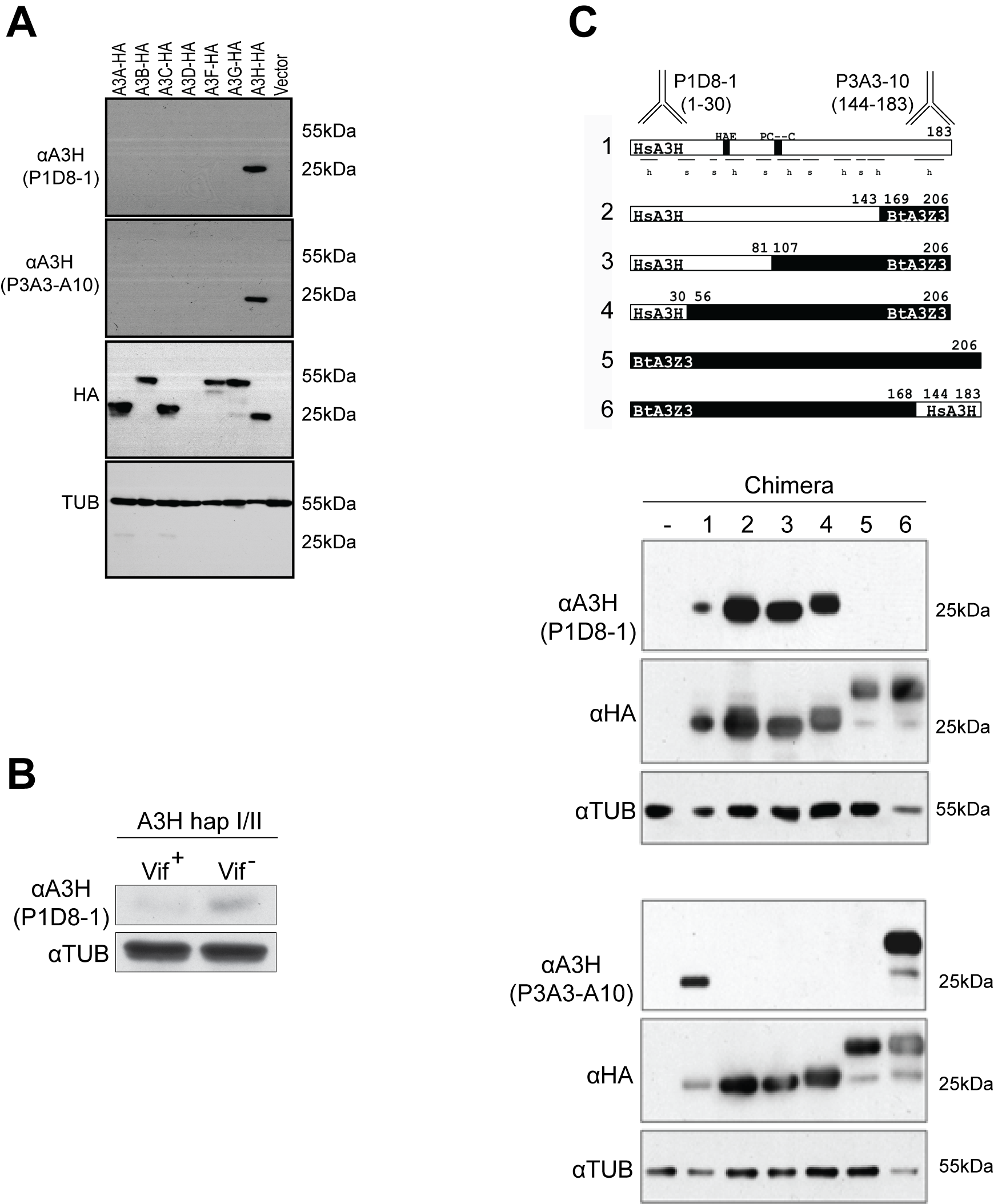

Supplement: Figure S1 — Characterization of mouse monoclonal antibodies specific to human APOBEC3H. A) Immunoblots demonstrating the specificity of the A3H monoclonal antibodies P1D8-1 and P3A3-A10 in 293T cells transiently expressing the indicated A3-HA protein. B) Immunoblots of A3H and tubulin expression levels in primary T lymphocytes 9 days after infection with Vif proficient or deficient viruses. A3D-HA expressed poorly in this experiment (donor 25). C) Schematics of human A3H hap II (open box), cow A3Z3 (black box), and A3H/A3Z3 chimeric derivatives. The epitopes for mouse monoclonal antibodies P1D8-1 and P3A3-A10 are shown. Immunoblots of 293T cells transiently transfected with the indicated human/cow chimeric A3H/A3Z3 constructs. Monoclonal antibodies P1D8-1 and P3A3-A10 recognize distinct N- and C-terminal epitopes, respectively. (TIF) [file pgen.1004761.s001.tif]

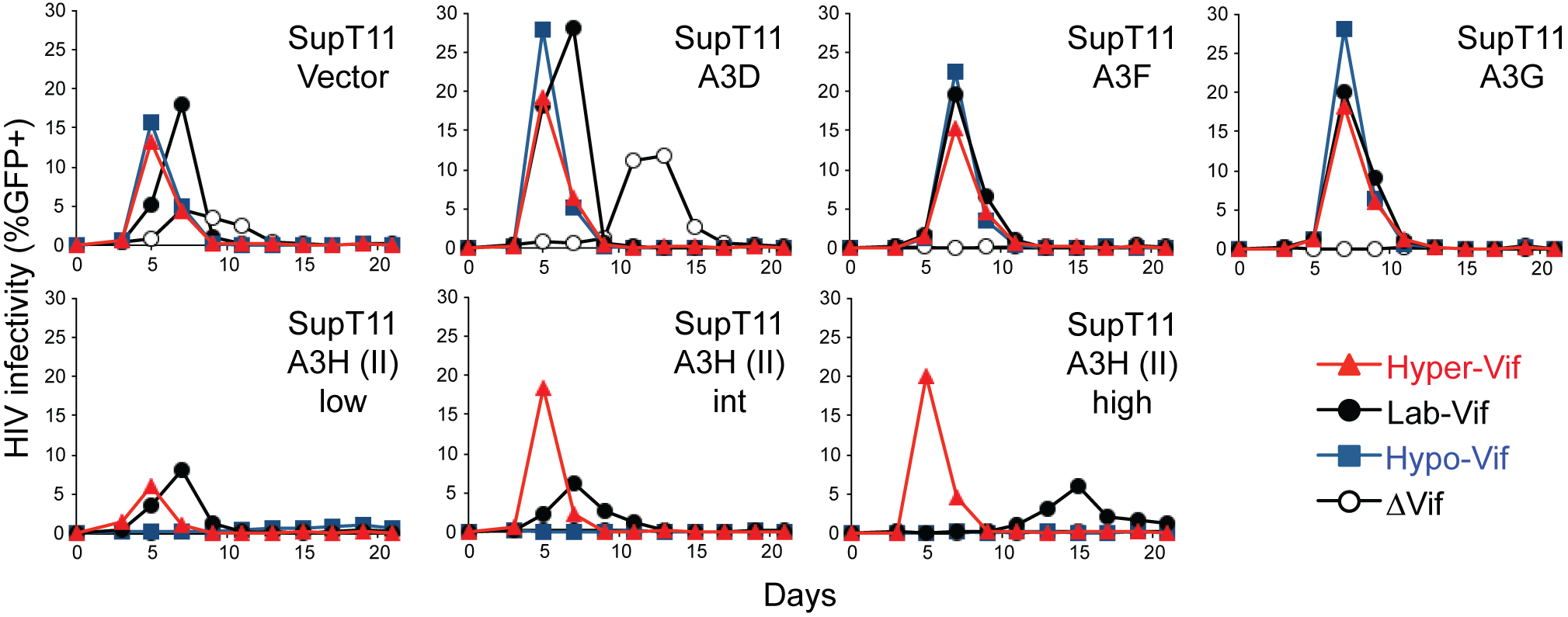

Supplement: Figure S2 — HIV-1 Vif separation-of-function molecular/viral probes. HIV-1 spreading infection kinetics for the indicated viruses on A3-expressing SupT11 cells lines described in Figure 3B. Data are reproduced here from Figure 3C but plotted on a common Y-axis scale. (TIF) [file pgen.1004761.s002.tif]

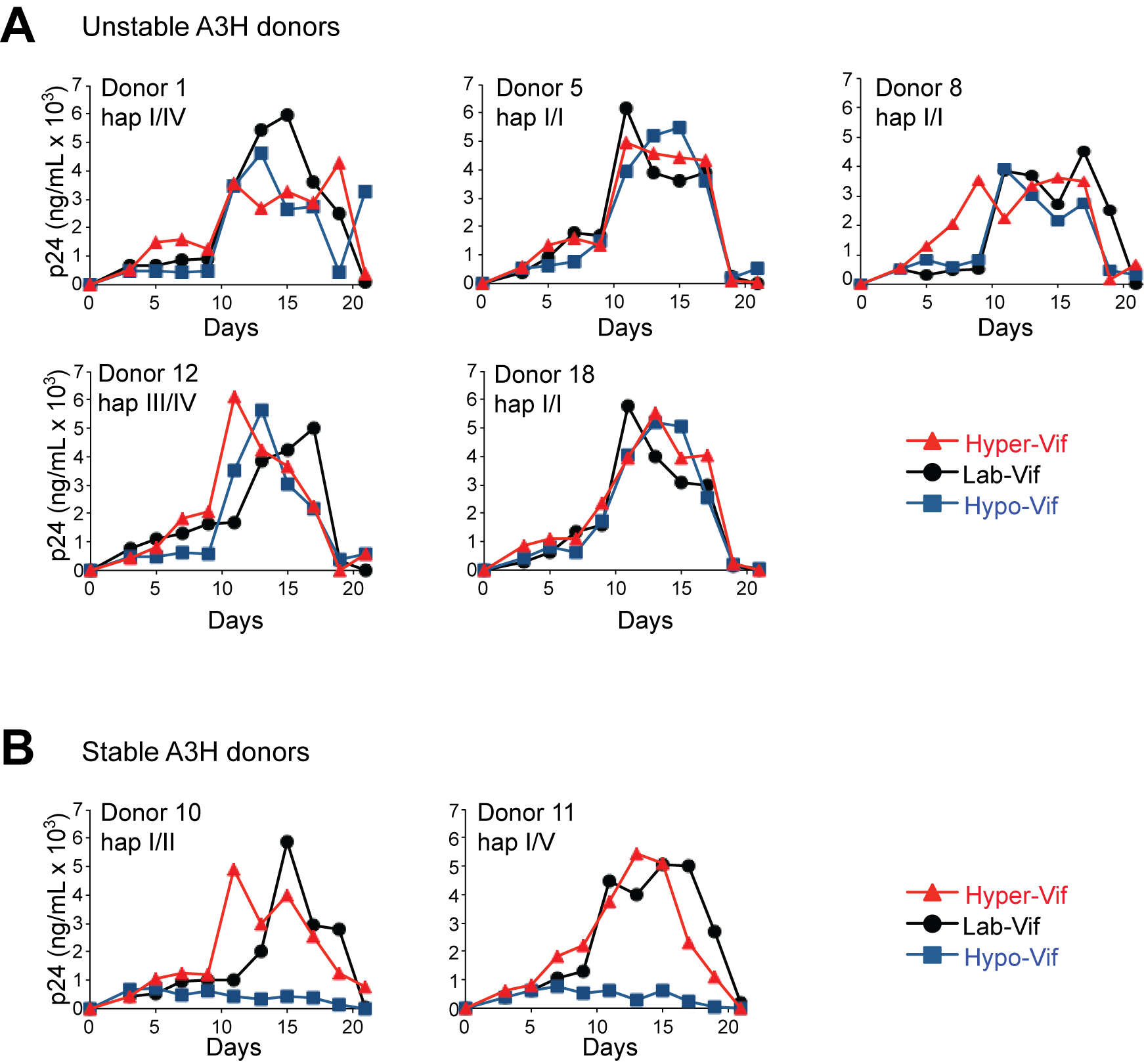

Supplement: Figure S3 — Stable APOBEC3H inhibits HIV-1 replication in primary T lymphocytes. A) HIV-1 replication kinetics of the hyper-, lab-, and hypo-Vif variants in CD4+ T lymphocytes from 5 healthy donors encoding the unstable A3H haplotype indicated (donors 1, 5, 8, 12, and 18). B) HIV-1 replication kinetics of the hyper-, lab-, and hypo-Vif variants in CD4+ T lymphocytes from 2 healthy donors heterozygous for the indicated allele of stable A3H (donors 10 and 11). We were not able to identify a second haplotype V donor and therefore have not been able to determine whether the lab-Vif phenotype of donor 11 is reproducible. (TIF) [file pgen.1004761.s003.tif]

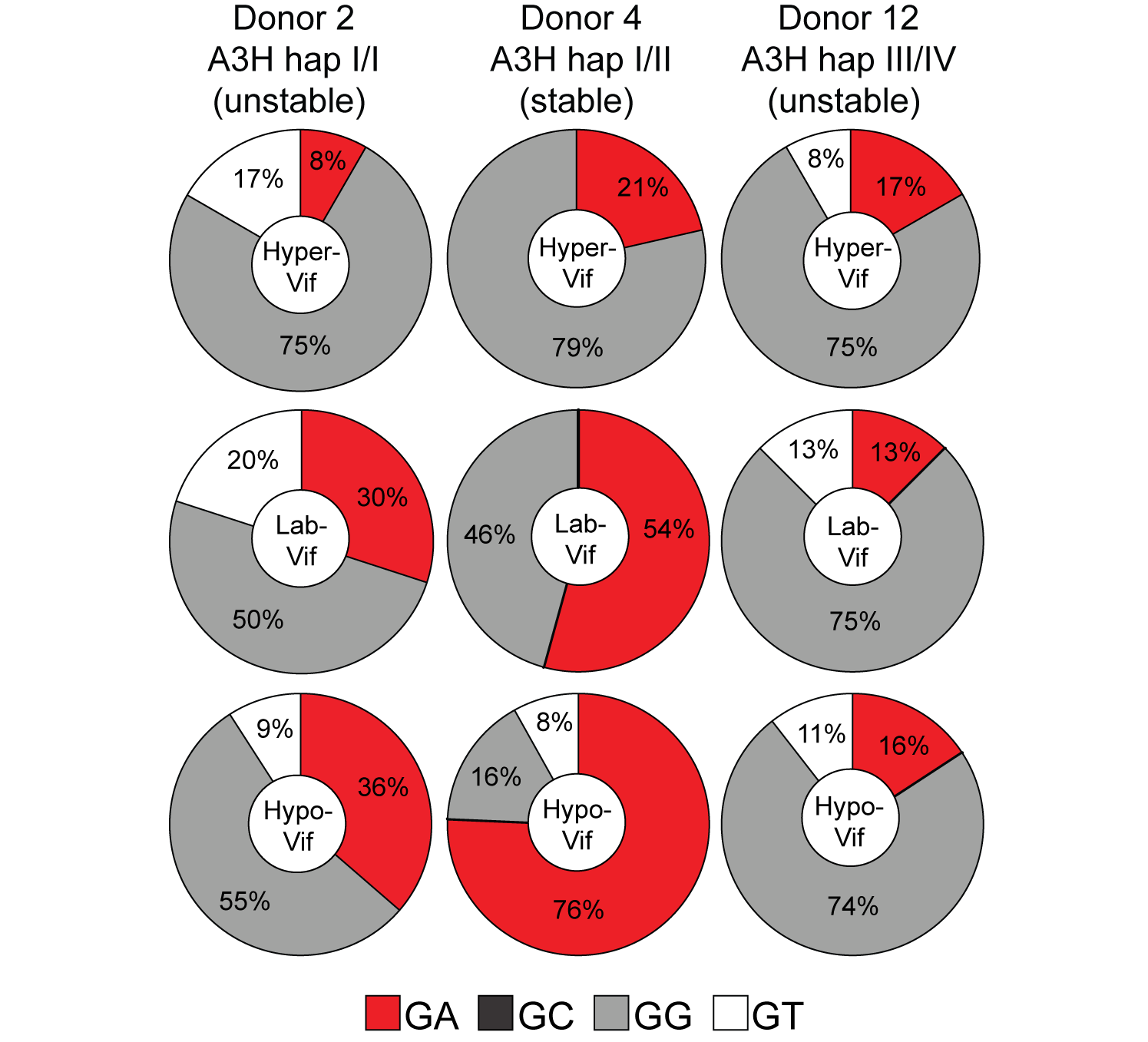

Supplement: Figure S4 — Stable APOBEC3H alleles inflict GA-to-AA hypermutations in viruses encoding hypo-Vif variants. HIV-1 G-to-A mutation profiles of the hyper-, lab-, and hypo-Vif proviruses originating from primary T lymphocytes with the indicated A3H haplotype (donors 2, 4, and 12). GA-to-AA mutations characteristic of A3H activity are shown in red. (TIF) [file pgen.1004761.s004.tif]

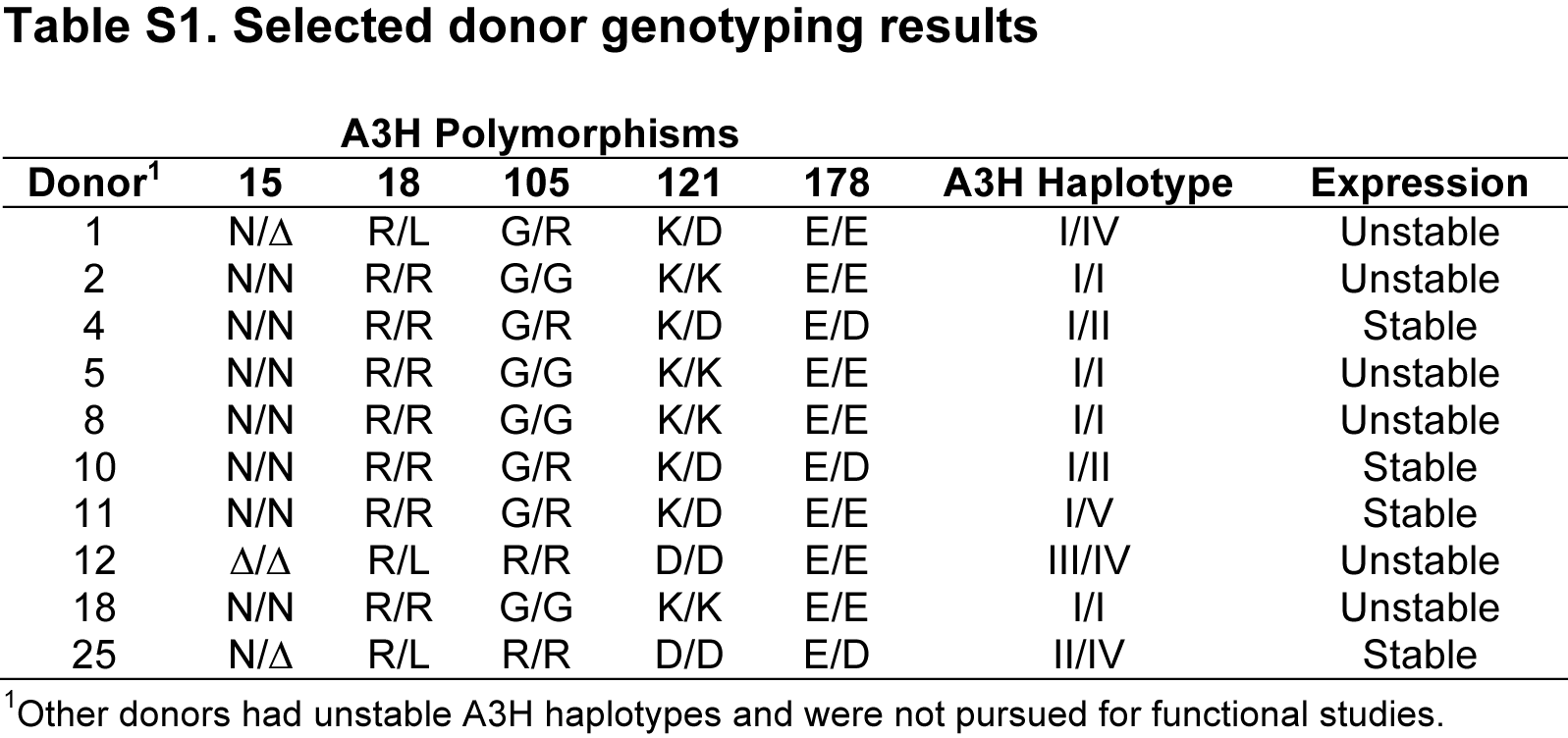

Supplement: Table S1 — Selected donor genotyping results. (TIF) [file pgen.1004761.s005.tif]

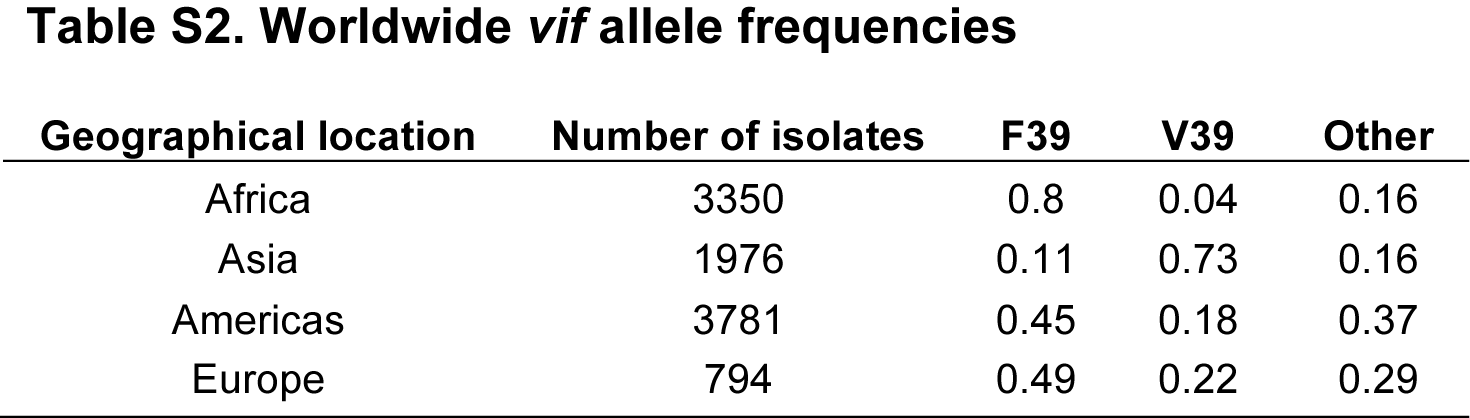

Supplement: Table S2 — Worldwide vif allele frequencies. (TIF) [file pgen.1004761.s006.tif]

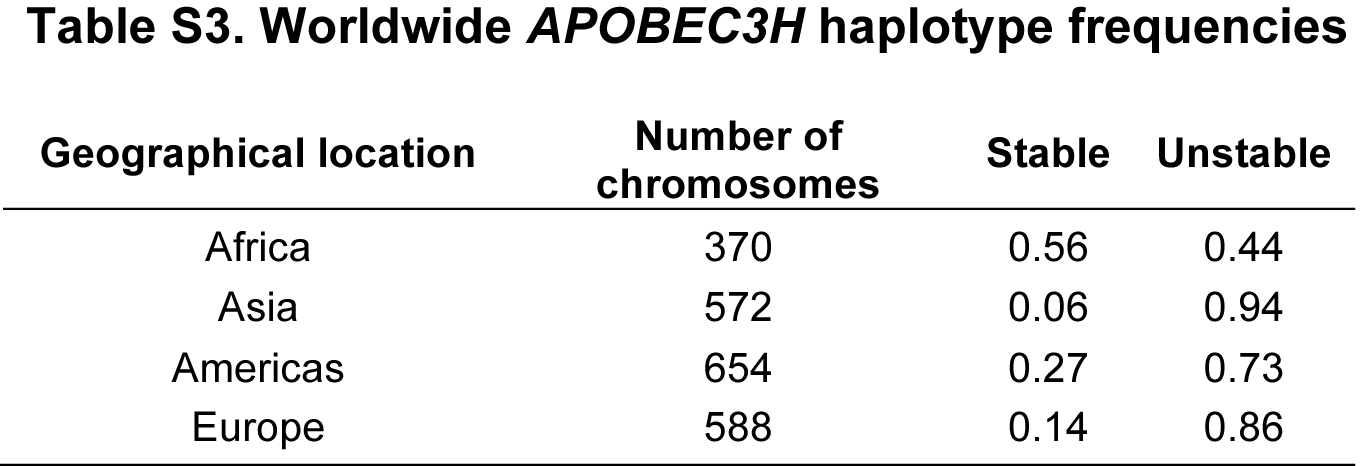

Supplement: Table S3 — Worldwide APOBEC3H haplotype frequencies. (TIF) [file pgen.1004761.s007.tif]
